# Editorial: Endometriosis: pathogenesis, diagnosis and treatment, volume II

**DOI:** 10.3389/fendo.2023.1334596

**Published:** 2023-12-18

**Authors:** Simone Ferrero, Mainak Dutta

**Affiliations:** ^1^ DINOGMI, University of Genova, Genova, Italy; ^2^ IRCCS Ospedale Policlinico San Martino, Genova, Italy; ^3^ Department of Biotechnology, Birla Institute of Technology and Science (BITS) Pilani-Dubai Campus, Academic City, Dubai, United Arab Emirates

**Keywords:** endometriosis, estrogen, pathogenesis, hypoxia, genetics, DNA methylation, microbiome, diagnosis

Endometriosis is a chronic, multifactorial, estrogen-dependent inflammatory disease characterised by the presence of endometriotic tissue outside the uterus, and it affects 6-10% of reproductive-age women (1). It causes pain symptoms (dysmenorrhea, deep dyspareunia, chronic pelvic pain) and infertility, thus significantly impairing quality of life. The gold standard for diagnosis of endometriosis is laparoscopy, followed by histological confirmation of the biopsy sample. Although non-invasive techniques, including ultrasonography and magnetic resonance imaging, may allow the detection of endometriosis (2, 3), diagnosing this condition is still challenging, and a non-invasive biomarker is not available (2). In addition, the etiology of the disease remains unclear.

Several studies included in this Research Topic tried to elucidate mechanisms involved in the pathogenesis of endometriosis. Identifying molecular pathways may also help develop diagnostic tools and identify potential pharmacological targets.

Endometriosis is typical of the reproductive age, and it usually regresses after menopause; however, it may recur if hormone replacement therapy is administered. Estrogens have a pivotal role in the pathogenesis of endometriosis because of their proliferative and inflammatory properties. Several studies included in this Research Topic investigated the role of estrogens in the pathogenesis of endometriosis. A review by Mercorio et al. described the endocrine microenvironment of endometriotic lesions. The review highlighted that the enzymatic pathways leading to locally increased synthesis of estrogens in endometriosis involve aromatase, 17β-hydroxysteroid dehydrogenase type 1, type 2 and type 5, steroid sulfatase, and estrogen sulfotransferase. A retrospective study by Emond et al. evaluated whether estradiol and its biologically active metabolites are differentially associated with endometriosis. The study included 209 women with endometriosis and 115 without endometriosis. Higher 2OH-3MeO-estrone was linked to an increased risk of endometriosis. Patients with ovarian endometriosis had enhanced 2-hydroxylation with higher 2MeO-estrone and 2OH-estrone levels. Abdominal, pelvic and back pain symptoms were also linked to higher 2OH-3MeO-estrone levels. Another review by Yuan et al. assessed the impact of several factors on estrogen-mediated epithelial-mesenchymal transition in the emergence of several diseases in the female reproductive tract, primarily endometriosis. Inhibition of estrogen biosynthesis may be a future target of endometriosis treatment (3).

According to the “retrograde menstruation theory”, when endometrial cells fall from the uterine cavity into the peritoneal cavity, they face severe hypoxic stress. A review by Zhou et al. evaluated the role of hypoxia-induced unfolded protein response in regulating the development of endometriotic lesions.

El Idrissi et al. investigated protein networks most strongly associated with the symptomatology of endometriosis. The authors found that biological pathways involving interleukin and/or cytokine signalling were linked to endometriosis-related symptoms.

It is well-known that genetic factors contribute to the pathogenesis of endometriosis. In this Research Topic, Bae et al. studies pathways underlying the pathogenesis of endometriosis by investigating differentially expressed genes in patients with endometriosis and healthy controls. One hundred eighteen differentially expressed genes were identified (79 upregulated and 39 downregulated) by integrating publicly available datasets. The authors validated the identified genes via immunohistochemical analysis of tissues obtained from patients with endometriosis and controls. They found that TLR4/NF-κB and Wnt/frizzled signalling pathways, as well as estrogen receptors, may be involved in the progression of endometriosis.

DNA methylation is essential in the regulation of gene expression. Changes in DNA methylation have been associated with altered gene expression in the endometrium, and they may contribute to the pathogenesis of endometriosis. A study by Lei et al. investigated candidate genes of endometriosis through integrated analysis of genome-wide gene expression and DNA methylation profiles. Eutopic and ectopic endometrial tissues were obtained from patients with ovarian endometriomas. Genome-wide methylation profiling identified 17551 differentially methylated loci, with 9777 hypermethylated and 7774 hypomethylated loci.

The microbiome may have a role in the development of endometriosis. A review by Uzuner et al. included in this Research Topic evaluated the role of the microbiome in the formation and progression of endometriosis via inflammatory pathways. The dysbiosis seen in endometriosis is thought to be both causative and a consequence of the pathogenesis. Gut, peritoneal fluid and female reproductive tract microbiota have been studied to understand if there are any microbiome signatures specific to endometriosis that can be used as a non-invasive test of the disease. Theoretically, manipulating the microbiome may also help treat endometriosis.

Endometriosis is associated with a decrease in ovarian reserve (4), and surgical treatment of endometriomas may further decrease ovarian function (5). A retrospective study by Liu et al. investigated the association between different ovarian reserves and reproductive and perinatal outcomes in patients with endometriosis. The study revealed that although patients with endometriosis with normal ovarian reserve and high ovarian reserve had increased reproductive outcomes, patients with endometriosis with diminished ovarian reserve still had an acceptable live birth rate and a similar cumulative live birth rate with available oocytes. Moreover, patients with normal ovarian reserve and high ovarian reserve might not exhibit a decreased risk of abnormal perinatal outcomes, except for gestational diabetes mellitus.

We want to express our profound gratitude to all the authors who contributed to this Research Topic by publishing their best works.

## Author contributions

SF: Writing – original draft. MD: Writing – review & editing.

## References

[1] Ferrero S Arena E Morando A Remorgida V. Prevalence of newly diagnosed endometriosis in women attending the general practitioner. Int J Gynaecol Obstet (2010) 110(3):203–7. doi: 10.1016/j.ijgo.2010.03.039 20546747

[2] Nisenblat V Bossuyt PM Shaikh R Farquhar C Jordan V Scheffers CS. Blood biomarkers for the non-invasive diagnosis of endometriosis. Cochrane Database Syst Rev (2016) 2016(5):CD012179. doi: 10.1002/14651858.CD012179 27132058 PMC7076288

[3] Barra F Romano A Grandi G Facchinetti F Ferrero S. Future directions in endometriosis treatment: discovery and development of novel inhibitors of estrogen biosynthesis. Expert Opin Investig Drugs (2019) 28(6):501–4. doi: 10.1080/13543784.2019.1618269 31072144

[4] Tian Z Zhang Y Zhang C Wang Y Zhu HL. Antral follicle count is reduced in the presence of endometriosis: a systematic review and meta-analysis. Reprod BioMed Online (2021) 42(1):237–47. doi: 10.1016/j.rbmo.2020.09.014 33168492

[5] Zhang Y Zhang S Zhao Z Wang C Xu S Wang F. Impact of cystectomy versus ablation for endometrioma on ovarian reserve: a systematic review and meta-analysis. Fertil steril (2022) 118(6):1172–82. doi: 10.1016/j.fertnstert.2022.08.860 36334993

